# Poisson’s ratio of individual metal nanowires

**DOI:** 10.1038/ncomms5336

**Published:** 2014-07-07

**Authors:** Eoin K. McCarthy, Allen T. Bellew, John E. Sader, John J. Boland

**Affiliations:** 1 School of Chemistry and Centre for Research on Adaptive Nanostructures and Nanodevices (CRANN), Trinity College Dublin, Dublin 2, Ireland; 2 Department of Mathematics and Statistics, The University of Melbourne, Victoria 3010, Australia

## Abstract

The measurement of Poisson’s ratio of nanomaterials is extremely challenging. Here we report a lateral atomic force microscope experimental method to electromechanically measure the Poisson’s ratio and gauge factor of individual nanowires. Under elastic loading conditions we monitor the four-point resistance of individual metallic nanowires as a function of strain and different levels of electrical stress. We determine the gauge factor of individual wires and directly measure the Poisson’s ratio using a model that is independently validated for macroscopic wires. For macroscopic wires and nickel nanowires we find Poisson’s ratios that closely correspond to bulk values, whereas for silver nanowires significant deviations from the bulk silver value are observed. Moreover, repeated measurements on individual silver nanowires at different levels of mechanical and electrical stress yield a small spread in Poisson ratio, with a range of mean values for different wires, all of which are distinct from the bulk value.

## Abstract (web summary)

Obtaining accurate mechanical data for nanowires is difficult and calls for complex methodology. Here, the authors report the design of a new lateral atomic force microscope set-up for electromechanically measuring the Poisson's ratio of nanowires with a high degree of accuracy.

## Graphical abstract

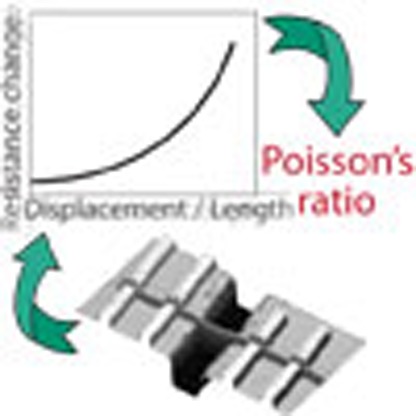

Poisson’s ratio, *ν*, is a fundamental mechanical parameter that describes the ratio of the lateral to the longitudinal strain of a material under mechanical load. The precise value of the Poisson’s ratio is controlled by the material’s microstructure. Greaves *et al.*^1^, reported on the importance of correlating the underlying crystal structure to observed values of *ν*. For macroscopic isotropic materials, the Poisson’s ratio *ν* is strictly limited between −1 and 0.5. However, for anisotropic materials there are no such bounds and for crystalline solids *ν* can vary widely depending upon which axis is being strained^2^. Since nanoscale materials can exhibit a range of different structures—from polycrystalline to single crystals with different growth directions—wide variations in *ν* might be expected for a given nanomaterial. The increased role of surface stress in nanoscale materials may also be expected to modify *ν* even in the case of polycrystalline nanomaterials. In general, *ν* is a difficult parameter to measure even in macroscopic materials and is usually derived from separate measurements of the shear and Young’s moduli. Recently, Signorello *et al.*^3^, introduced a method to estimate *ν* using photoluminescence measurements—an approach that is applicable only to direct semiconductors and requires a special cladding to prevent quenching of the luminescence. Despite significant computational activity^4^,^5^,^6^, at present there is no straightforward experimental method to measure the Poisson’s ratio *ν* of nanoscale materials.

Here we describe a general approach for the measurements of *ν* in wire systems. Our approach is applicable to macro, micro and nanoscale materials, although it is most readily implemented for metal nanowires (NWs). This method involves the mechanical manipulation of a double-clamped wire, while a simultaneous four-point electrical resistance measurement is performed using a precision source-measure unit that enables the simultaneous measurement of the current through and the voltage across the wire while the wire undergoes mechanical manipulation.

## Results

### Experimental method and application to macroscopic wires

**Figure 1:**
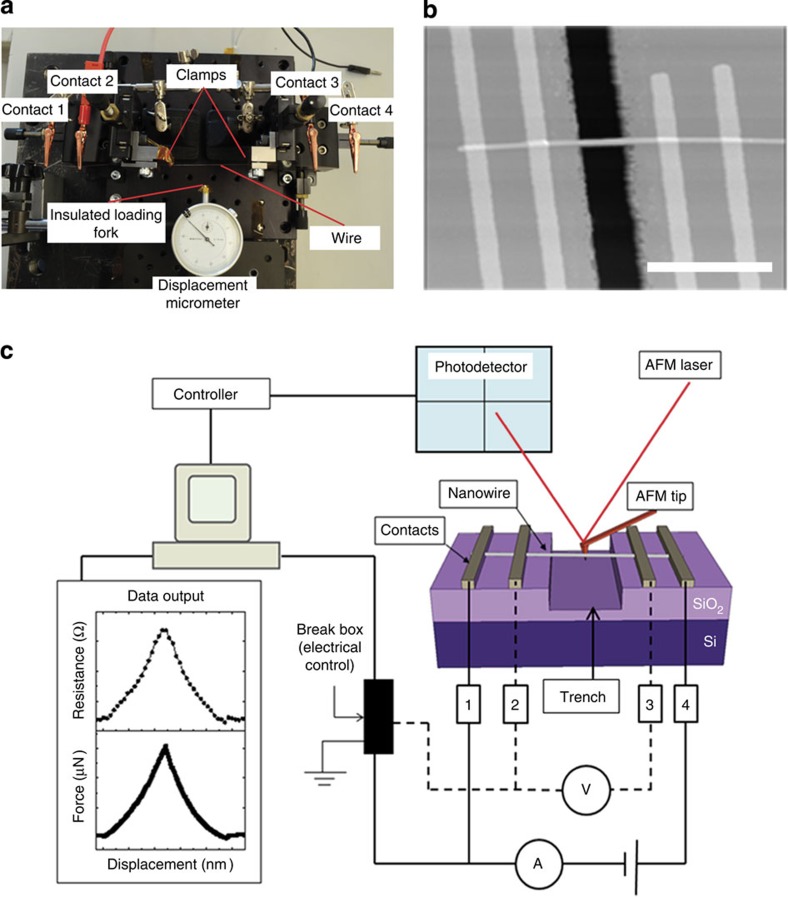
Experimental design for macroscopic and nanoscale wires. (**a**) Photograph of the bulk wire three-point bending and four-point electrical apparatus. The wire is mechanically clamped and then loaded using a calibrated micrometre. Electrical contact is achieved via four crocodile clips. (**b**) AFM image of an individual suspended AgNW with four-contact electrodes (scale bar, 4 μm). (**c**) Schematic representation of the nanowire electromechanical experiment. The AFM allows for accurate mechanical characterization by lowering the AFM tip below the axis of the nanowire, into a predefined trench, and laterally driving the tip in the *x*–*y* plane perpendicular to the nanowire long axis until the AFM tip loads the nanowire. The lateral signal from the AFM tip torsional motion during nanowire loading is measured in unison with the four-point resistance giving resistance– and force–displacement curves. A full description of the experimental method can be found in the methods section.

**Figure 2:**
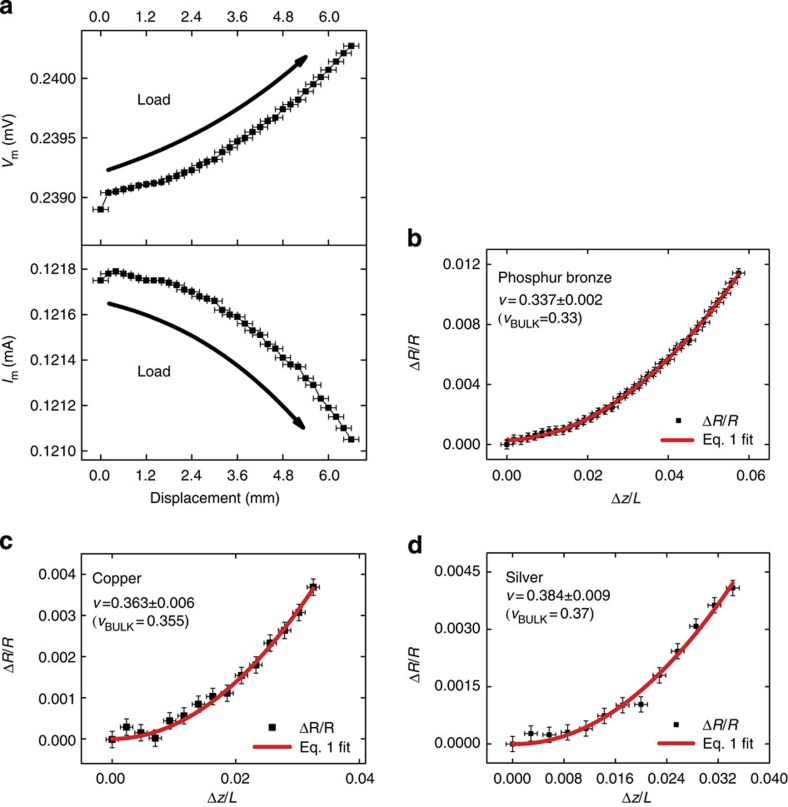
Macroscopic wire experimental data. (**a**) Measured current, *I*_m_, and measured voltage, *V*_m_, as a function of displacement for a 125-μm diameter phosphor bronze wire. (**b**) The relative change in resistance as a function of Δ*z/L* for an individual 125-μm diameter phosphor bronze wire. The fit to equation (1) is given by the red curve showing the model that can be applied from macro to nanoscale materials. (**c**) The relative change in resistance as a function of Δ*z/L* for an individual 100-μm diameter Cu wire. The fit to equation (1) is given by the red curve. (**d**) The relative change in resistance as a function of Δ*z/L* for an individual 50-μm diameter Ag wire. The fit to equation (1) is given by the red curve. In **a**, **b** and **c** the *x*–*y* error bars originate from the resistance and micrometre experimental uncertainty. Error in *ν* is the standard error (one sigma) from the fit of equation (1). (The typical current density applied is ~\n246 A m^−2^).

The experimental set-up is shown in Fig. 1a and its nanoscale implementation in Fig. 1c. The former consists of a double-clamped wire (0.125–0.05 mm in diameter) that undergoes a three-point bend by the controlled displacement of the middle of the clamped wire length using a calibrated precision micrometre. Electrical measurements are made using four alligator-clip contacts attached to the wire as shown; the current is sourced at the outer contacts by applying a fixed source voltage, while the voltage drop across the wire and the current through the wire (which changes as the wire resistance increases due to manipulation) are measured. The latter are then combined to provide a measurement of the wire resistance. Figure 2a shows the measured change in voltage, *V*_m_, and measured current, *I*_m_, through a (macroscopic) 125 μm diameter phosphorous bronze wire, as a function of wire displacement. The mechanical manipulation is completely elastic and the measured values return to the original level when the wire is unloaded. Note that deviations in the data are frequently observed at very small displacements as the electrically isolated manipulation fork gains purchase on the wire. Figure 2b shows the corresponding resistance change for this polycrystalline phosphor bronze wire as a function of lateral displacement, which is the result of the increase in length and reduction in cross-section of the wire during elastic manipulation.

The observed relative change in resistance (Δ*R/R*) as a function of the normalized displacement per unit length (Δ*z/L*) is analyzed in terms of a new model that accounts for bending and tensile deformations. The Δ*R/R* term is experimentally derived from the resistance change divided by the initial resistance prior to mechanical manipulation. The Δ*z/L* term is given by the NW displacement at the point of loading, Δ*z*_center_, divided by the original length, *L*, of the clamped wire. Our model predicts:


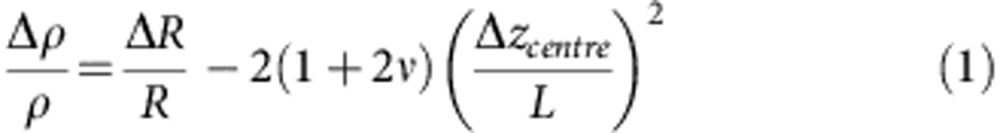


where *ρ* is the wire resistivity and *ν* is Poisson’s ratio; the full derivation is provided in Supplementary Discussion. Equation (1) is valid when the displacement is greater than the wire radius, consistent with the conditions in the present experiments (see Fig. 2). Here we demonstrate our method for metal wires since in the elastic regime Δ*ρ* is expected to be zero for metals, and a fit of equation (1) to the data can be used to determine the value of *ν.*

Using this approach we determine *ν* for bulk (macroscopic) cylindrical metal wires (phosphor bronze, copper and silver) and obtained values that are in excellent agreement with known values for these isotropic materials. Figure 2b–d show the fit of equation (1) to the experimental data for each wire. We find the value of Poisson’s ratio to be *ν*=0.337±0.002 (Standard deviation) for phosphor bronze (bulk=0.33), 0.363±0.006 for copper (bulk=0.355), and 0.384±0.009 for silver (polycrystalline bulk value is 0.37). In all instances the experimental values for Poisson’s ratio *ν* agree closely with the corresponding bulk value, which validates both the experimental approach and the model used herein (the full data set is presented in Supplementary Table 1).

### Application to nanoscale wires

To extend this approach to NWs we use the set-up shown in Fig. 1c, which consists of a closed-loop Asylum MFP-3D atomic force microscope (AFM) combined with a home-built probe station capable of making accurate four-contact electrical measurements. NW mechanical properties are measured using an AFM lateral three-point bending manipulation technique developed previously^7^,^8^,^9^,^10^. This allows the full spectrum of mechanical properties to be measured, ranging from elastic to plastic response and failure, and during which a simultaneous measurement of the resistance change of the NW is recorded. The wire is placed over a trench to accurately define the pinning length and to eliminate friction with the substrate during mechanical manipulation, AFM image in Fig. 1b (see Supplementary Figs 3 and 4, and Supplementary notes 1 and 2)).

**Figure 3:**
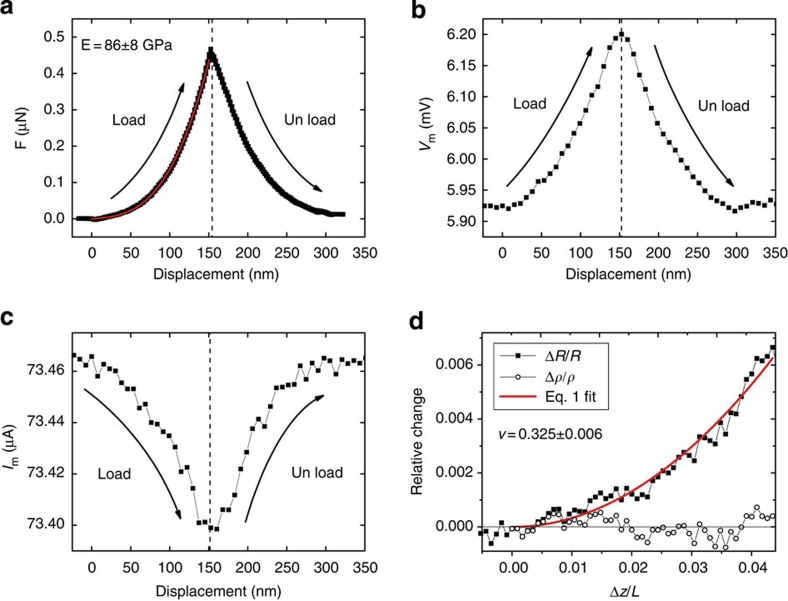
Nanoscale wire experimental data. (**a**–**c**) Force, measured current and measured voltage as a function of displacement. The red curve in **a** is a fit to the generalized mechanical model^10^. (**d**) The relative change in resistance and resistivity as a function of Δ*z/L* for an individual 40-nm radius NiNW. See Supplementary Figs 8 and 9 showing the change in resistance and resistivity as a function of Δ*z/L* for multiple NiNW and AgNW tested. (Typical source voltage of 25 mV).

The relative resistance change, Δ*R/R*, as function of Δ*z/L* is studied for nickel NWs (NiNW) and silver NWs (AgNW) of diameters between 50 and 90 nm. The force–displacement curve for each NW is recorded simultaneously with the four-point measured current, *I*_m_, and measured voltage, *V*_m_ (a representative AgNW IV curve is shown in Supplementary Fig. 2). Figure 3a–c shows typical force *F*, *V*_m_ and *I*_m_ curves as a function of displacement for an individual metallic NW. In all experiments the NW is elastically loaded and unloaded, as shown in the force–displacement curve in Fig. 3a. The curve is non-linear and symmetric about the dashed line, indicating full elastic recovery after unloading. The Young’s modulus is extrapolated from the red line fit to the generalized model^10^ (see Supplementary Figure 1 and Supplementary Discussion), yielding 86±8 GPa in this case. The expected force dependence on displacement is observed, with no plastic deformation. This is evident from the definite and sharp change in force as the AFM tip reverses direction from the loading to the unloading cycle. During loading, *V*_m_ (Fig. 3b) increases by ~\n4.5% to a maximum at the point of maximum load, and then recovers to the previous unloaded value. Conversely, *I*_m_ decreases by <1% during loading, Fig. 3c, to a minimum corresponding to the maximum load. The drop in *I*_m_ is due to the increased resistance of the wire during manipulation and the fixed source voltage employed in these experiments and is accompanied by an even larger increase in *V*_m_. Both *I*_m_ and *V*_m_ completely recover to their unloaded values. Measurement of *I*_m_ and *V*_m_ is then used to determine Δ*R/R* at each point during the manipulation. Interestingly, we do not observe an increase in resistance in the unloaded wire at the current densities employed here, see Supplementary Figs 5 and 6. Moreover the original resistance is always recovered after mechanical unloading, regardless of the current density or the electrical/mechanical cycling history. These results demonstrate that there are no irreversible changes in the wire and that the wire behaviour is electrically ohmic, that is, there is no current-induced heating of the NW (The temperature dependence of the resistance can be written as *R* (*T*+Δ*T*)=*R* (*T*) (1+α Δ*T*), where α~\n0.004 K^−1^ for metals). Given the NW resistance is typically 15 Ω, a temperature increase of Δ*T*=1 K will cause a resistance increase of 0.06 Ω, which is well above the 0.001 Ω sensitivity of our measurement (see Figs 2 and 3). Further details are presented in Supplementary Figs 5 and 6.

**Figure 4:**
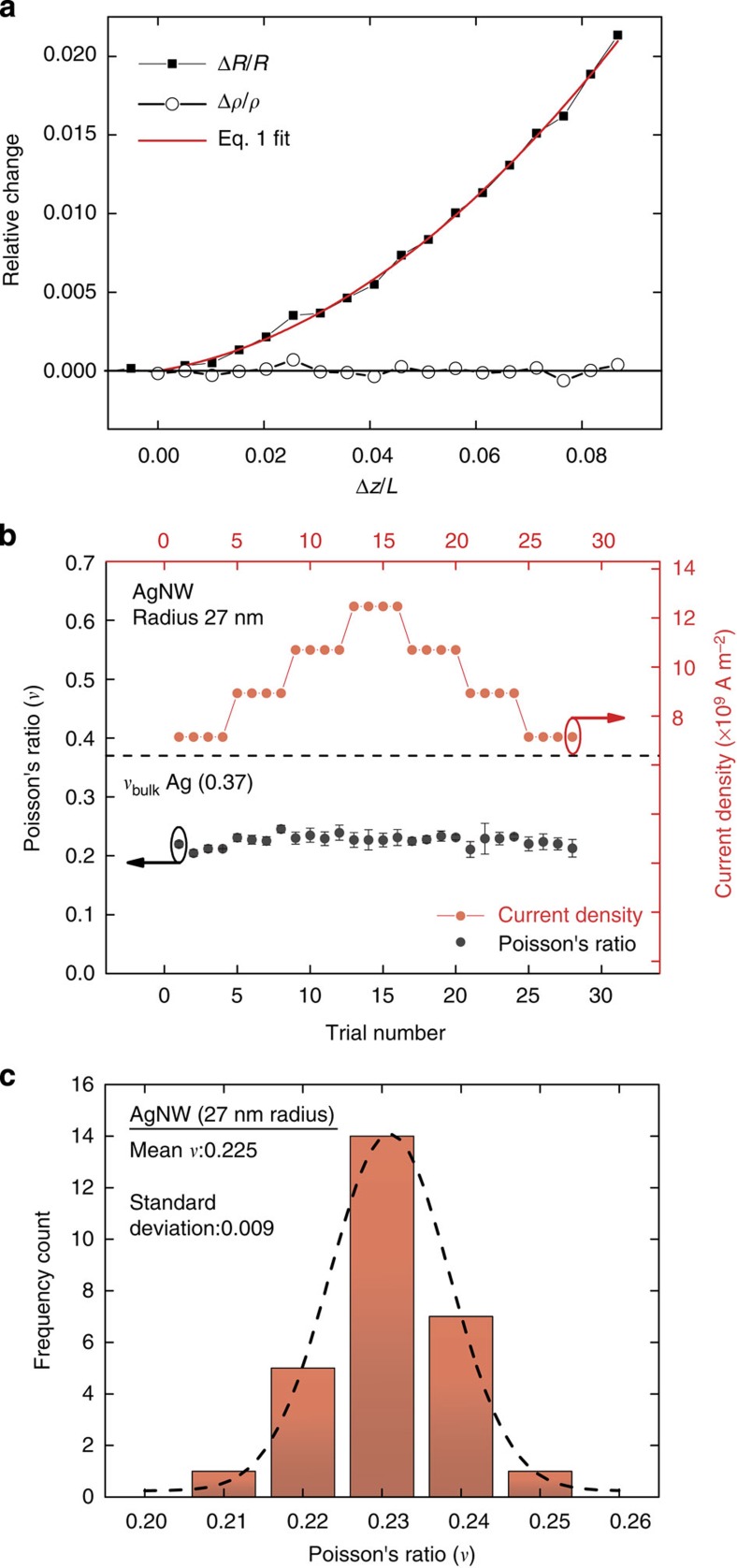
Detailed characterization on 27 nm AgNW. (**a**) A typical curve showing the relative change in resistance as a function of Δ*z/L* for an individual 27-nm radius AgNW (E=86±8 GPa). (**b**) Twenty-eight independent Poisson’s ratio measurements (grey filled symbols) on a 27-nm radius AgNW at increasing and decreasing current density levels. The red triangular stepped plot shows the current density conditions at which each of the corresponding Poisson’s ratio values are measured. The dashed line represents *ν* for isotropic bulk silver (0.37). (**c**) Histogram showing the mean value of Poisson’s ratio shown in **b** is 0.225 with a standard deviation of 0.009. The error bars in **b** are given by the standard fitting error of equation (1) to each Δ*R/R* versus Δ*z/L* curve, including the error in the length which introduces an uncertainty of <1.5%.

Figure 3d shows measured values of Δ*R/R* as a function of Δ*z/L* for a 40-nm radius NiNW, for which the quadratic dependence (filled squares) on Δ*z/L* predicted by equation (1) is clearly seen. We note that the NiNW data is noisy when compared with AgNWs, see Fig. 4a. This is due to the presence of an oxide and requires that individual contacts to these NiNWs have to be separately formed through a process known as resistive switching (see Supplementary Fig. 7). No effort is made to remove the surface oxide. Our model is still applicable since the surface oxide layer is very thin and its Young’s modulus is significantly less than that of Ni metal^11^ so that the oxide does not affect the wire deformation. Δ*ρ/ρ* is the residual following the fit of Δ*R/R* to the quadratic term in equation (1) and is shown as the black circles in Fig. 3d. As expected for a metal NW, *ρ* does not change with increasing Δ*z/L* so that the observed resistance change is fully accounted for by the increased NW length and the reduction in cross-section during loading. The Poisson’s ratio is determined from the quadratic coefficient to the fit of equation (1). In addition, this analysis provides an estimate of the gauge factor (GF), which is defined as the relative change in resistance as a function of strain, *ε*=Δ*L/L*=2(Δ*z/L*)^2^, so that from equation (1) we can write:


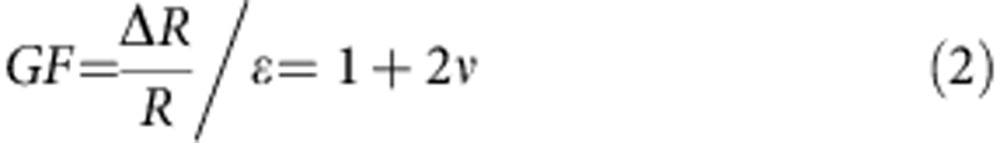


Figure 3d shows the measured value of *ν* for a 40-nm radius NiNW, which is found to be 0.325±0.006, in excellent agreement with the bulk value of 0.31. Several NiNWs (39–44 nm in radius) are analyzed using this same approach, resulting in values of *ν* shown in Table 1 (see Supplementary Fig. 8 for details). The average value of Poisson’s ratio is found to be 0.317±0.011, with very little spread in values among the wires, and with all wires exhibiting values close to the known bulk value of 0.31. The GF (both measured directly and evaluated using equation (2)) gives identical results with values of 1.635±0.058. Based on these data, the Poisson’s ratio and GF for a polycrystalline NiNW are essentially identical to those of a bulk macroscopic Ni wire.

We perform identical measurements on a range of AgNWs with radii between 27 and 32 nm. Figure 4a shows the measured values of Δ*R/R* as a function of Δ*z/L* for a 27-nm radius AgNW, and the quadratic dependence (filled squares) on Δ*z/L* predicted by equation (1). As expected, the behaviour is similar to that for NiNWs, both being metals, but the signal-to-noise ratio is greatly improved due to the absence of a surface oxide. Again, as expected for a metal there is no evident change in Δ*ρ/ρ* during deformation. The results for the range of AgNWs studied are summarized in Table 2. The literature bulk polycrystalline value of *ν* for Ag^12^ is 0.37 and the result for bulk single crystal Ag strained along the [110] direction is 0.36 (see below). In contrast to the tight spread in *ν* about the bulk isotropic value observed for the macroscopic wires in Fig. 2 and the NiNWs in Table 1, the AgNWs in Table 2 display large variations from the bulk value, with distinct (and repeatable) values of *ν* for different wires (the full data set is presented in Supplementary Table 1).

### Detailed measurement on an individual AgNW

To probe the origin of the different values of *ν* in Table 2, and to rule out any possibility of unexpected changes in the wire structure due to loading, we perform a detailed analysis on an individual 27 nm AgNW. This involves a series of experiments in which the same wire is mechanically manipulated while its resistance is measured at a series of increasing and decreasing current density steps. The Poisson’s ratio values determined from these experiments are shown in Fig. 4b. The mean Poisson ratio obtained by averaging over the 28 individual experiments is 0.225±0.009, significantly less than the bulk value, but consistent with the data in Table 2 obtained for other AgNWs. These experiments confirm that values of *ν* recovered from the present method are well defined, repeatable and independent of the current densities employed or the history of the wire. Figure 4c shows a histogram analysis of the data recorded from the 27 nm AgNW. A Gaussian distribution of measurement values about the mean is observed with a relative standard deviation of 4%, thus providing evidence of the technique’s robustness and ability to accurately determine Poisson’s ratio (the full data set is presented in Supplementary Table 1).

## Discussion

**Figure 5:**
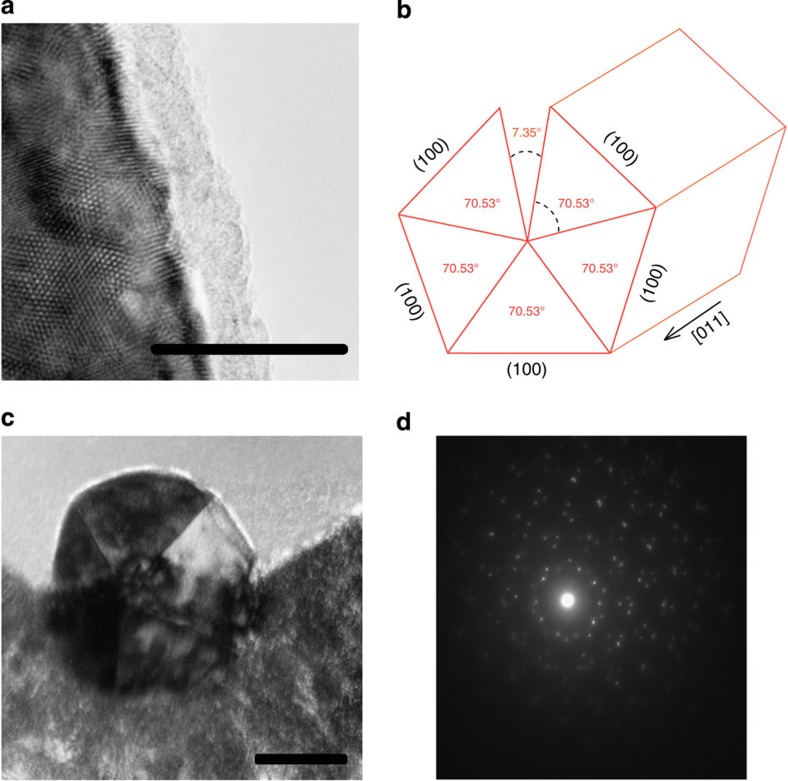
Transmission electron microscopy characterization of nickel and silver nanowire. (**a**) Transmission electron microscopy (TEM) image of NiNW showing polycrystalline microstructure. (**b**) Schematic of the pentagonally twinned structure indicating the 7.35° deficiency. (**c**) TEM image of AgNW cross-section displaying the fivefold pentagonal twinning. (**d**) Diffraction pattern taken from the wire shown in **c** where the fivefold twinning structure is evident. (Scale bars, 20 nm). A detailed analysis of the NW dimensions is presented in the Supplementary Figs 10 and 11.

To explain the different behaviours of NiNWs and AgNWs we consider the possible roles of current density and wire material structure. Both wires yield values of *ν* that are independent of current density. Since the value of *ν* must be sensitive to the wire’s material structure, this indicates that the wires do not undergo any change in material structure as a result of current flow. This conclusion is consistent with the fact that all measured properties of the wires (resistance, stiffness) are fully recovered even after the wires have been exposed to the highest current densities reported here. NiNWs yield values of *ν* that are close to the bulk value with similar values for NWs of different dimensions. On the other hand, AgNWs shows a large spread in values even though repeated measurements on the same wire are reproducible with high precision over a wide range of current densities. Together, these point to a fundamental difference in the material structure of AgNWs and NiNWs. The NiNWs used in this work are polycrystalline and similar in material structure to the macroscopic wires described in Fig. 2. This is clearly seen in the transmission electron microscopy image shown in Fig. 5a. In contrast, transmission electron microscopy analysis also shows that the AgNWs are single crystals that grow along the [110] direction with pentagonal {111} twin planes running down the entire wire length (see Supplementary Fig. 10).

While the Poisson’s ratio for isotropic/polycrystalline materials such as the NiNWs is bounded so that −1≤*ν*≤½, no such bounds exists for anisotropic materials such as the AgNWs considered in this work. Theoretical analysis typically considers the components of the transverse strain in specific perpendicular directions to the applied stress. Importantly, Baughman *et al.*^13^ showed that for a [110] applied stress in cubic materials, that is, identical to the direction of the applied stress in our AgNWs, the Poisson’s ratios in the 
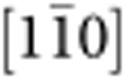
 and [001] directions are bounded between −1<*ν*<0 and 0<*ν*<2, respectively^13^. Using known elastic constants^14^, we calculate Poisson’s ratios of −0.094 and 0.82 in the two orthogonal directions transverse to the loading direction^13^, yielding a net Poisson’s ratio of 0.36 for a single crystal Ag specimen strained along the [110] direction^15^. Explanation for the different values of *ν* found for different AgNWs remains an open question. One possibility is that the measured variations in Poisson’s ratio are related to different twinning structures in the AgNWs. Pentagonally twinned NWs are known to grow under conditions that produce significant stress^16^. The five (111) bounded subunits that comprise the wire cannot completely fill space, resulting in a solid angle deficit of 7.35 degrees shown in Fig. 5b, which is overcome at the expense of incorporating lattice strain and or defects within these wires^17^. Moreover, it is known that the position of pentagonal twinning axis can vary from wire to wire leading to different degrees of stress relaxation^17^. Further theoretical and experimental work is required to connect these expected changes in microstructure to the observed variations in Poisson’s ratio. The enabling capacity to measure the Poisson’s ratio of NWs developed here is thus expected to be pivotal in addressing this and other important questions in nanoscale materials science.

In conclusion, we have introduced a method that provides a robust and precise measure of the Poisson’s ratio of individual metal NWs. This capability will enable new insights into the mechanical properties of nanoscale materials and will undoubtedly raise fundamental questions that will increase our understanding of these important materials. This approach will also enable researchers to evaluate the electromechanical performance of individual wires, their operation in nanoelectromechanical devices and the properties of flexible NW-based materials and composites.

## Methods

### Sample preparation

AgNWs and NiNWs are purchased from Seashell Technology ( http://www.seashelltech.com/) and Nanomaterials.it ( http://www.nanomaterials.it/), respectively. Samples for electromechanical experiments are prepared by drop casting a NW suspension on pre-patterned trenches on a SiO_2_ substrate. Trenches (250 nm deep) are fabricated using conventional ultra violet-lithography and CH_4_/Ar reactive-ion-etching (RIE). Contacting electrodes are defined by Electron-Beam-Lithography, with individual NWs contacted by four, 120-nm thick, electron beam evaporated silver electrodes.

### Mechanical characterization

All mechanical measurements are performed using 75-kHz rectangular cantilevers purchased from Budget sensors. A complete description of the tip calibration procedure is described extensively elsewhere^8^. An Asylum MFP-3D AFM equipped with a lateral lithography suite and closed *x*–*y* loop is used to elastically load the NWs in ambient conditions at room temperature. Prior to manipulation the NW long axis is aligned parallel to the cantilever axis to avoid slippage between the NW and the tip during mechanical deformation.

### Electrical characterization

Prior to electromechanical experiments individual NWs are electrically characterized using the standard four-point method on a stand-alone Karl Suss probe station supported by a Keithley 4200 SCS parameter analyzer. The resistance and resistivity values measured on the probe station and on the home-built electromechanical system (below) are in close agreement.

### Electromechanical measurements

Electromechanical experiments are performed on a home-built four-point electrical apparatus attached to an Asylum MFP-3D AFM. The electrical apparatus consists of a Keithley 6430 SMU and Keithley 2000 multimeter controlled via a National Instruments LabView interface. Connection to the sample is made via standard coaxial cabling to a home-built support frame in which a chip carrier is mounted. The electrodes contacting the NW on the fabricated SiO_2_ substrate are connected to a chip carrier by Au wire bonding using a standard wire bonder system. Importantly, the support frame sits flat on the AFM scanner with access to the cables from below. This set-up allows for the simultaneous measurement of the mechanical and electrical properties.

### AFM tip loading procedure

The loading cycle procedure is performed as follows: (1) the AFM tip loads the NW to a specific user defined displacement, (2) when the tip reaches this displacement, it reverses its direction and unloads the NW at the same velocity (20 nm s^−1^) as the initial loading, (3) this results in the inverted V shaped *f*–*d* curve seen throughout.

### Error analysis

The reported uncertainty in the measured values of *ν* is one standard deviation from the derived parameter, given by the fit of equation (1) to the data, derived using standard error propagation. The measured physical dimensions of the clamped length of the wire using AFM have <1.5% uncertainty; this uncertainty is determined by comparison between AFM and scanning electron microscope images. AFM length measurements are necessarily a lower bound due to tip convolution.

## Author contributions

E.K.M. wrote the manuscript, designed and carried out the experiments. A.T.B assisted with NiNW preparation. J.E.S. developed the mathematical models used to describe the data. J.J.B. wrote the manuscript and led the overall effort. E.K.M, J.E.S and J.J.B discussed the results and contributed to writing of the manuscript.

## Additional information

**How to cite this article:** McCarthy, E.K. *et al.* Poisson’s ratio of individual metal nanowires. *Nat. Commun.* 5:4336 doi: 10.1038/ncomms5336 (2014).

## Supplementary Material

Supplementary Information Supplementary Figures 1-11, Supplementary Table 1, Supplementary Notes 1-6, Supplementary Discussion and Supplementary References

## Figures and Tables

**Table 1:** Measured poisson’s ratios for NiNWs.

**NW radius (nm)**	**Poisson’s ratio,** ***ν***
39	0.335±0.014
40	0.325±0.006
42	0.316±0.004
43	0.306±0.024
44	0.304±0.008

NW, nanowire.

*ν* is the Poisson’s ratio. Uncertainty is one standard deviation.

**Table 2:** Measured poisson’s ratios for AgNWs.

**NW radius (nm)**	**Poisson’s ratio,** ***ν***
27	0.225±0.009
30	0.191±0.003
32	0.601±0.023
39	0.294±0.015

NW, nanowire.

*ν* is the Poisson’s ratio. Uncertainty is one standard deviation.
