# Supplementary material for: Poisson’s ratio of individual metal nanowires
Source: Nat Commun. 2014 Jul 7;5:4336. doi: 10.1038/ncomms5336 (PMC4102115; doi:10.1038/ncomms5336)
Supplement: Supplementary Information — Supplementary Figures 1-11, Supplementary Table 1, Supplementary Notes 1-6, Supplementary Discussion and Supplementary References [file ncomms5336-s1.pdf]

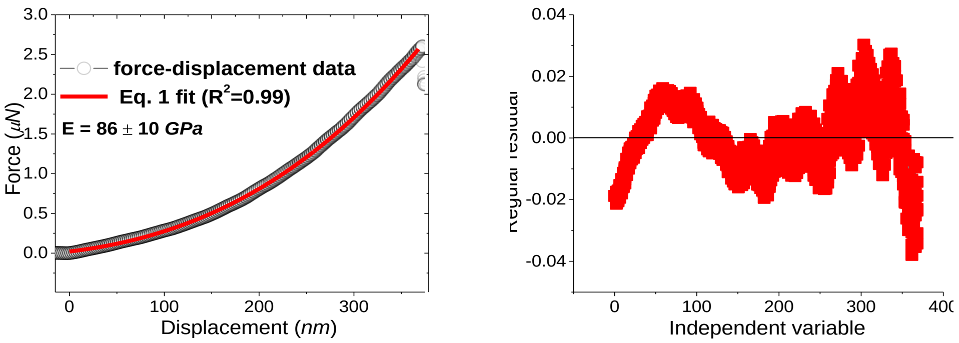

(a) (b)

Supplementary Figure 1: (**a**) Typical pentagonal Ag NW force-displacement curve, the Young's modulus is 86 ± 10 GPa, calculated from the red fit of equation 1 to the grey data, (**b**) residuals for the fit of the generalised model to the data.

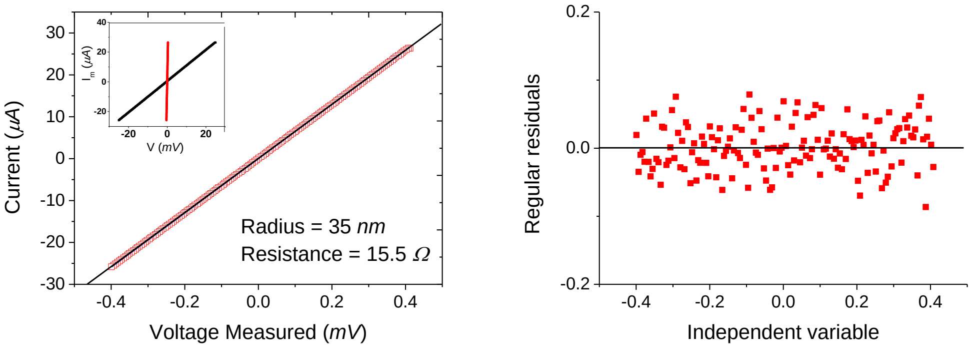

(a) (b)

Supplementary Figure 2: (**a**) Typical pentagonal AgNW 2- and 4-point IV curve. The linear fit is given by the black line through the red data and used to calculate the resistance using Ohms law, (**b**) residuals of the linear fit to extrapolate the resistance.

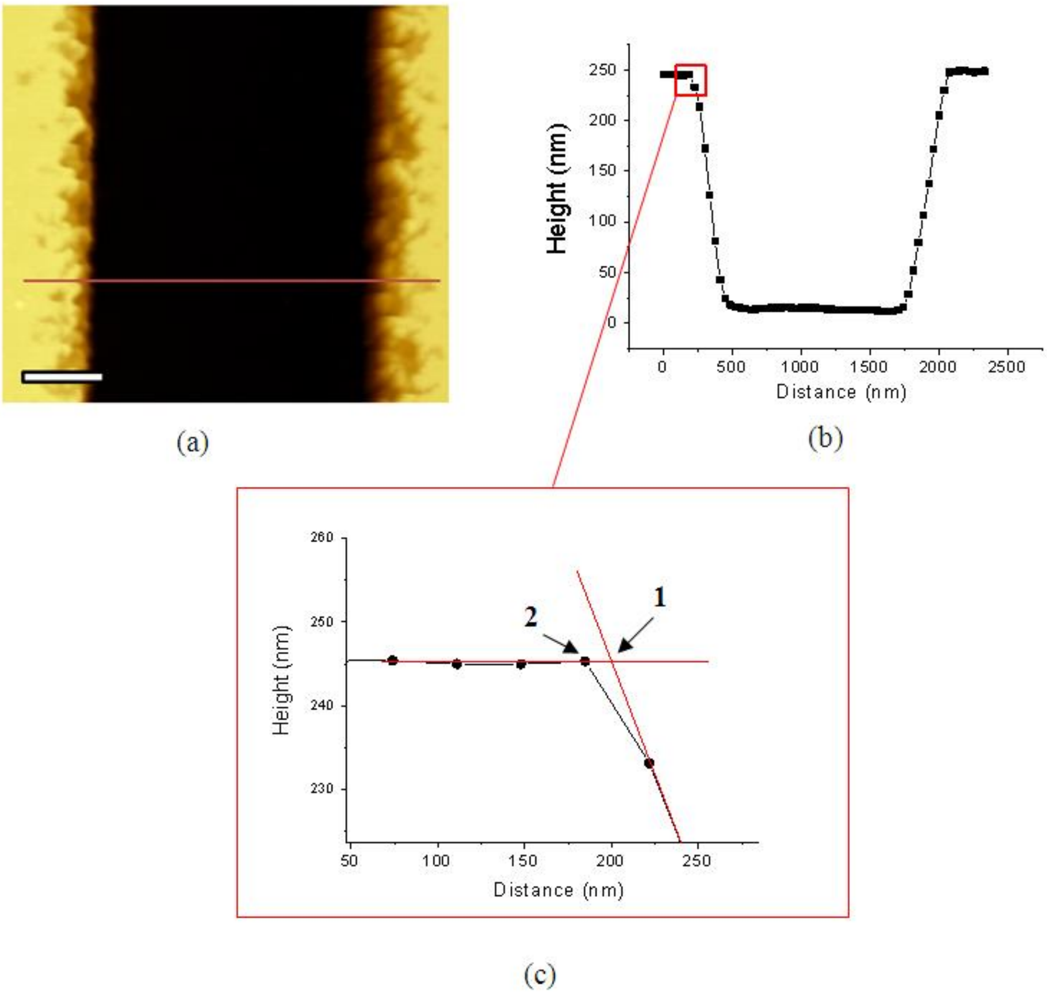

Supplementary Figure 3: (**a**) AFM image of trench region showing the bottom of the trench as well as both edges. (**b**) line profile taken across the red line shown in **a**. (**c**) Enlarged plot of the area with the red square in **b**. The distance between points 1 and 2 is the AFM measurement accuracy. See supplementary note 1 for details.

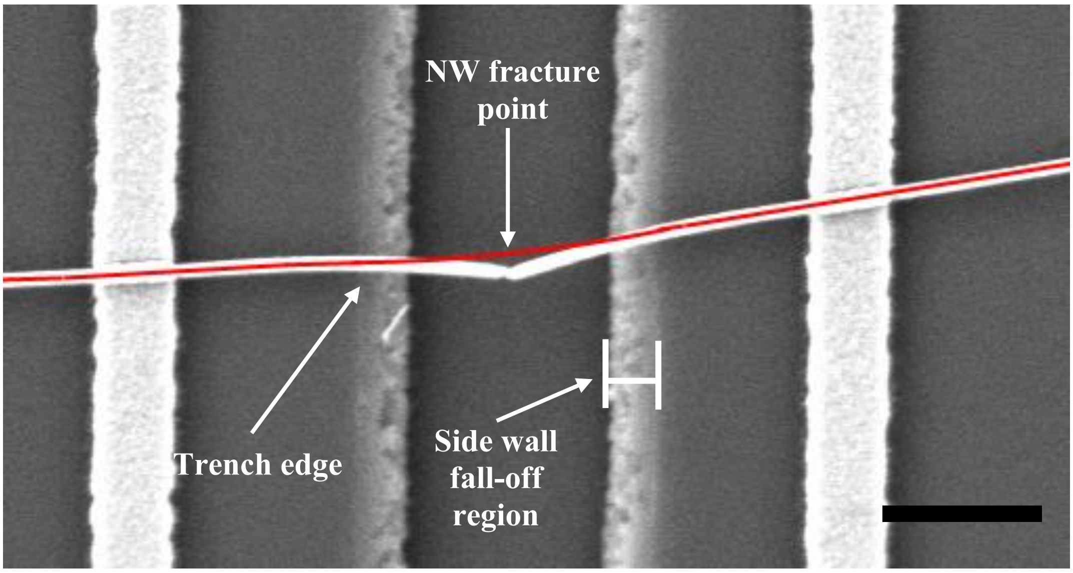

Supplementary Figure 4: SEM image of a fractured AgNW showing no surface slippage from the trench edge at either side (scale bar is 1 μm). See supplementary note 2 for details.

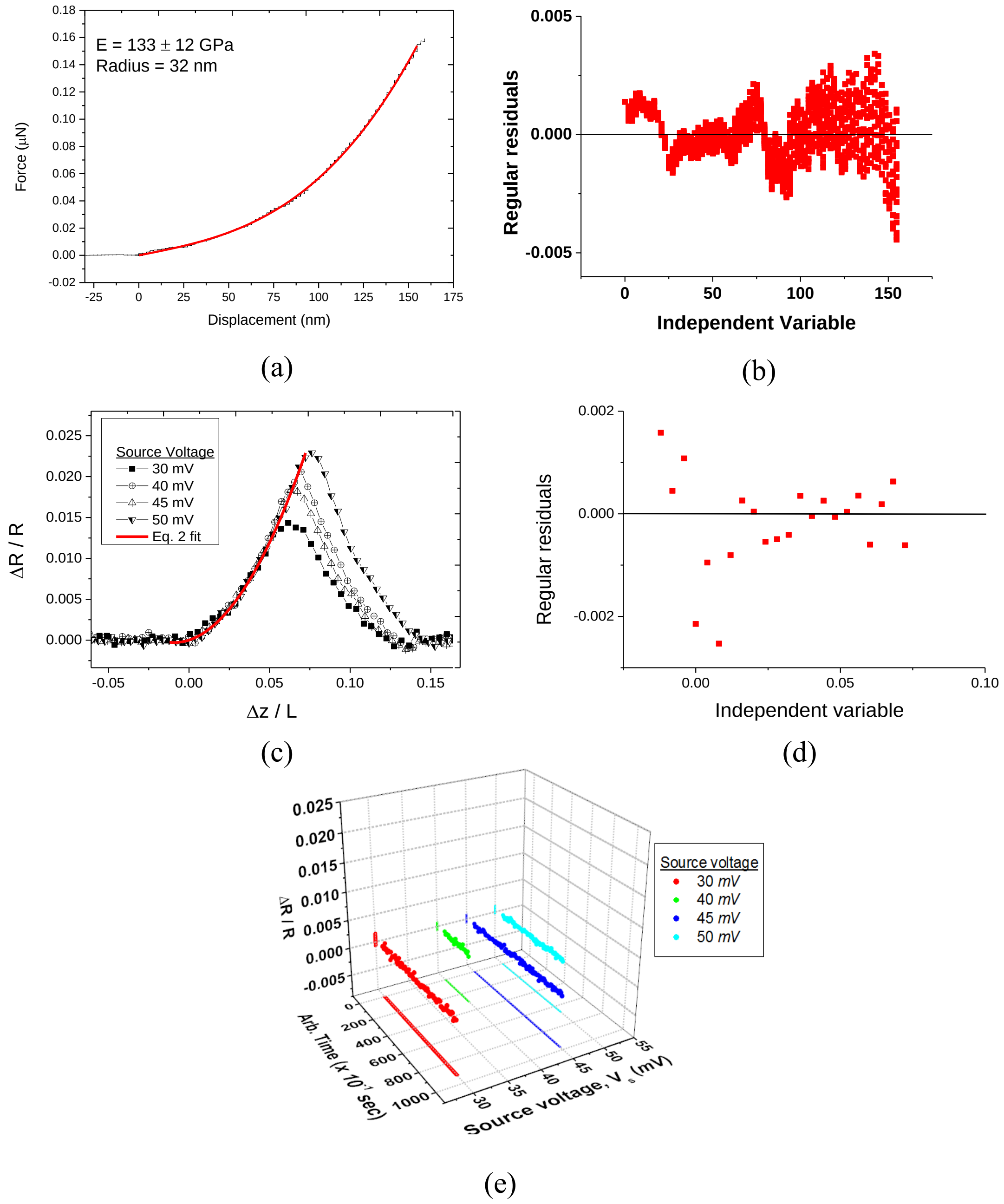

Supplementary Figure 5: (**a**) 32 nm Ag NW force-displacement curve, the Young's modulus is 133 ± 12 GPa, calculated from equation 1, (**b**) residuals for the fit of the mechanical model to the data, (**c**) The change in resistance as a function of strain for an individual 32 nm radius AgNW under increasing current loads, the red curve is a fit to the model. Note the curves have been shifted so that the rising edges overlap. (**d**) Residuals to the fit in **c** at 50 mV source voltage. (**e**) The relative change in resistance as a function of time and source voltage for a 32 nm Ag NW, showing no resistance dependence on time or source voltage. See supplementary note 3 for further details.

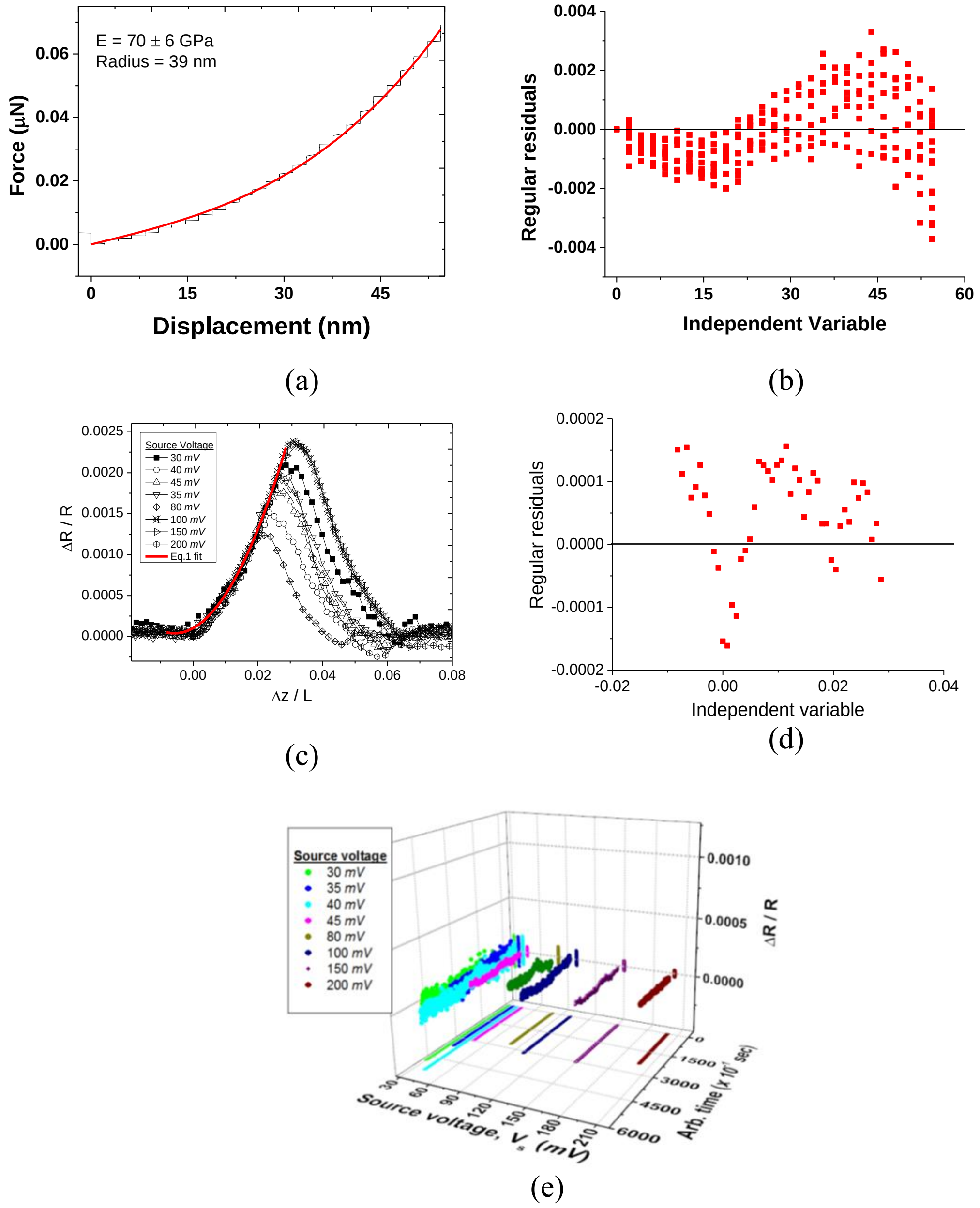

Supplementary Figure 6: (**a**) 39 nm Ag NW force-displacement curve, the Young's modulus is 70 ± 6 GPa, calculated from the generalised mechanical model, (**b**) residuals to the fit, (**c**) The change in resistance as a function of strain for an individual 39 nm radius AgNW under increasing current loads, the red curve is a fit to the model, (**d**) residuals to the fit. (**e**) The relative change in resistance as a function of time and source voltage for a 39 nm Ag NW, showing no resistance dependence on time or source voltage. See supplementary note 3 for further details

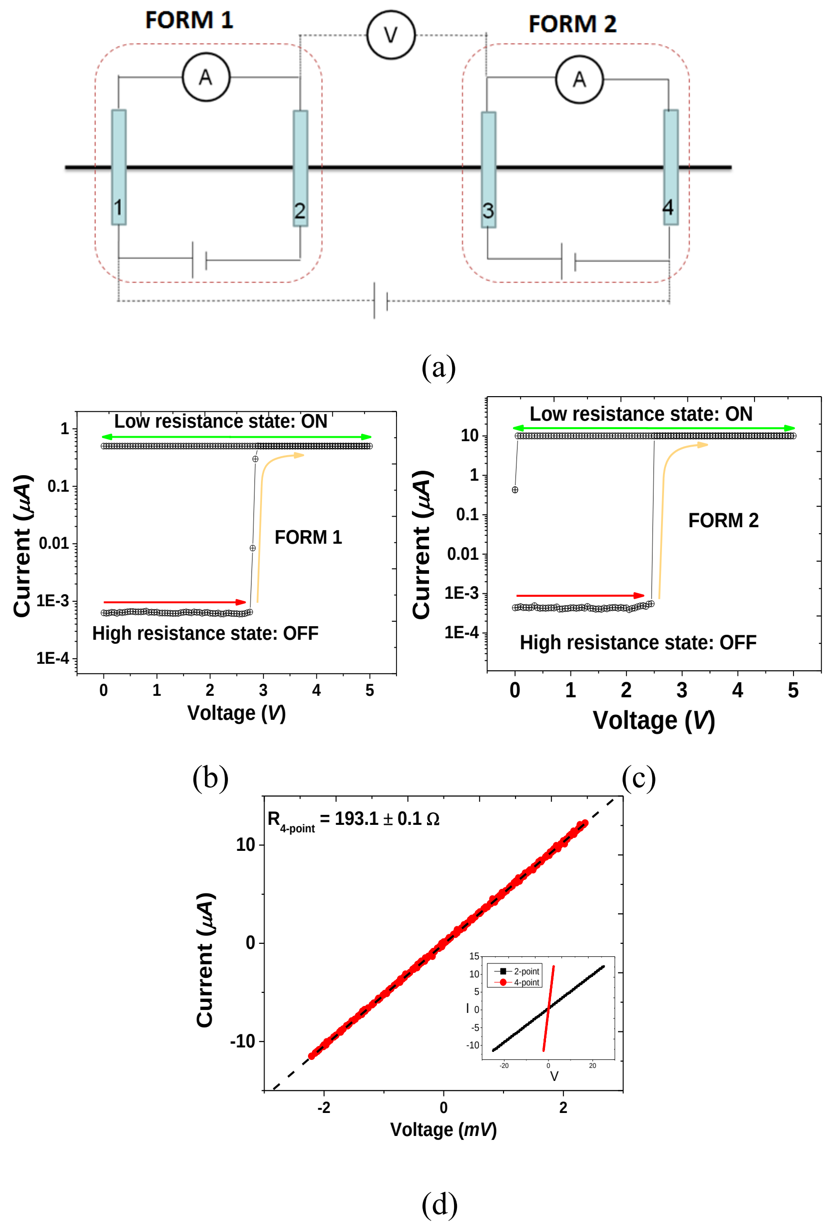

Supplementary Figure 7: (**a**) Schematic of an individual nanowire contacted with four blue contacts. The NW is first formed across electrodes 1 and 2 (FORM 1), then across electrodes 3 and 4 (FORM 2). The 4-point measurement was then performed by sourcing the voltage and measuring the current the outer electrodes 1 and 4, and measuring the voltage was measured across the inner electrodes 2 and 3. (**b**, **c**) IV curves for the FORM 1 and FORM 2 steps respectively, showing the characteristic change from the high resistance to low resistance state in an RS system (**d**) 4-point IV curve for an individual NiNW. Inset is the 2- and 4-point curves. See supplementary discussion for further details.

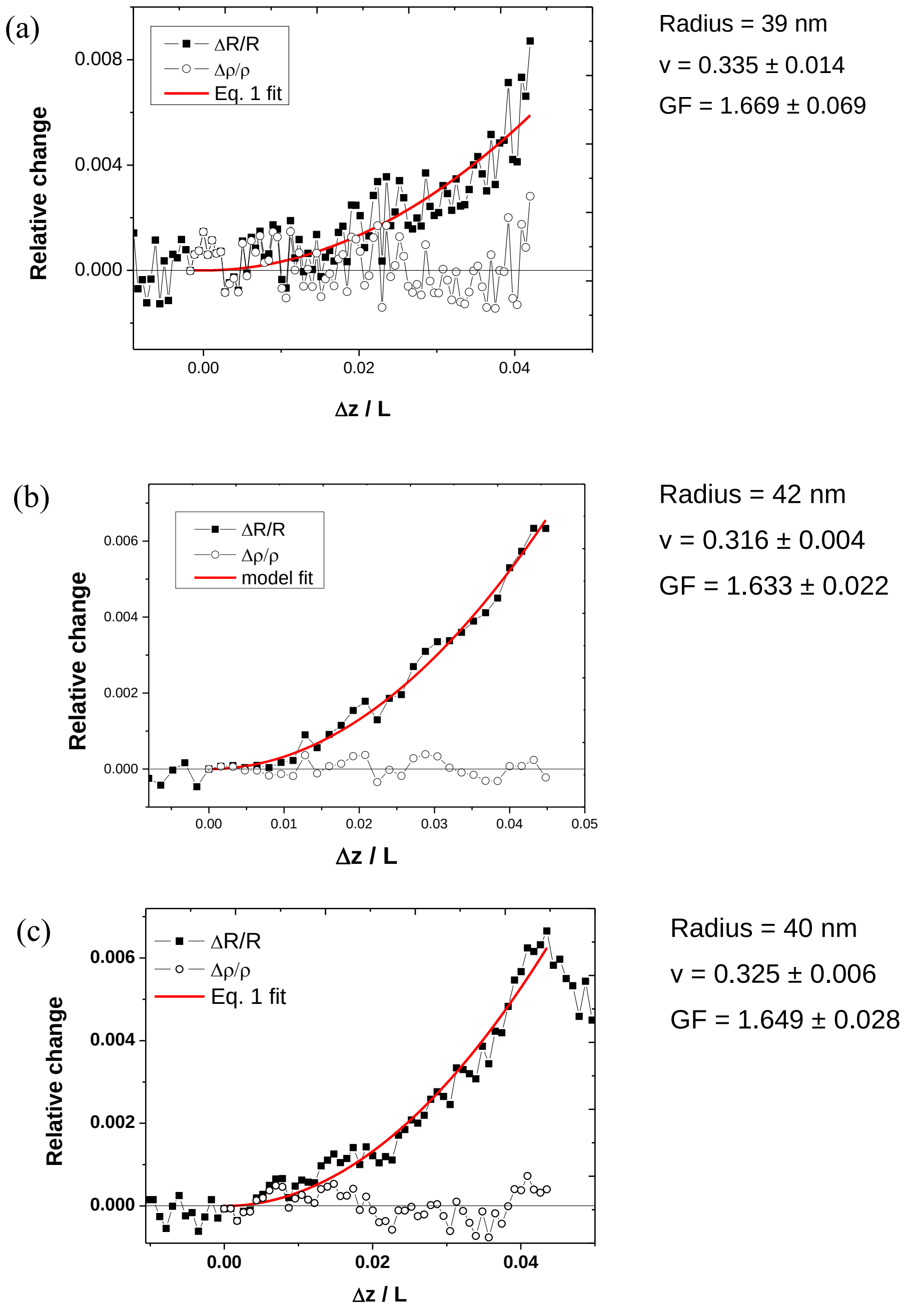

Supplementary Figure 8: Relative change in resistivity and resistance for NiNWs of radii (**a**) 39 nm, (**b**) 42 nm and (**c**) 40 nm. Poisson's ratio and gauge factor given for each NiNW. See supplementary note 4 for further details.

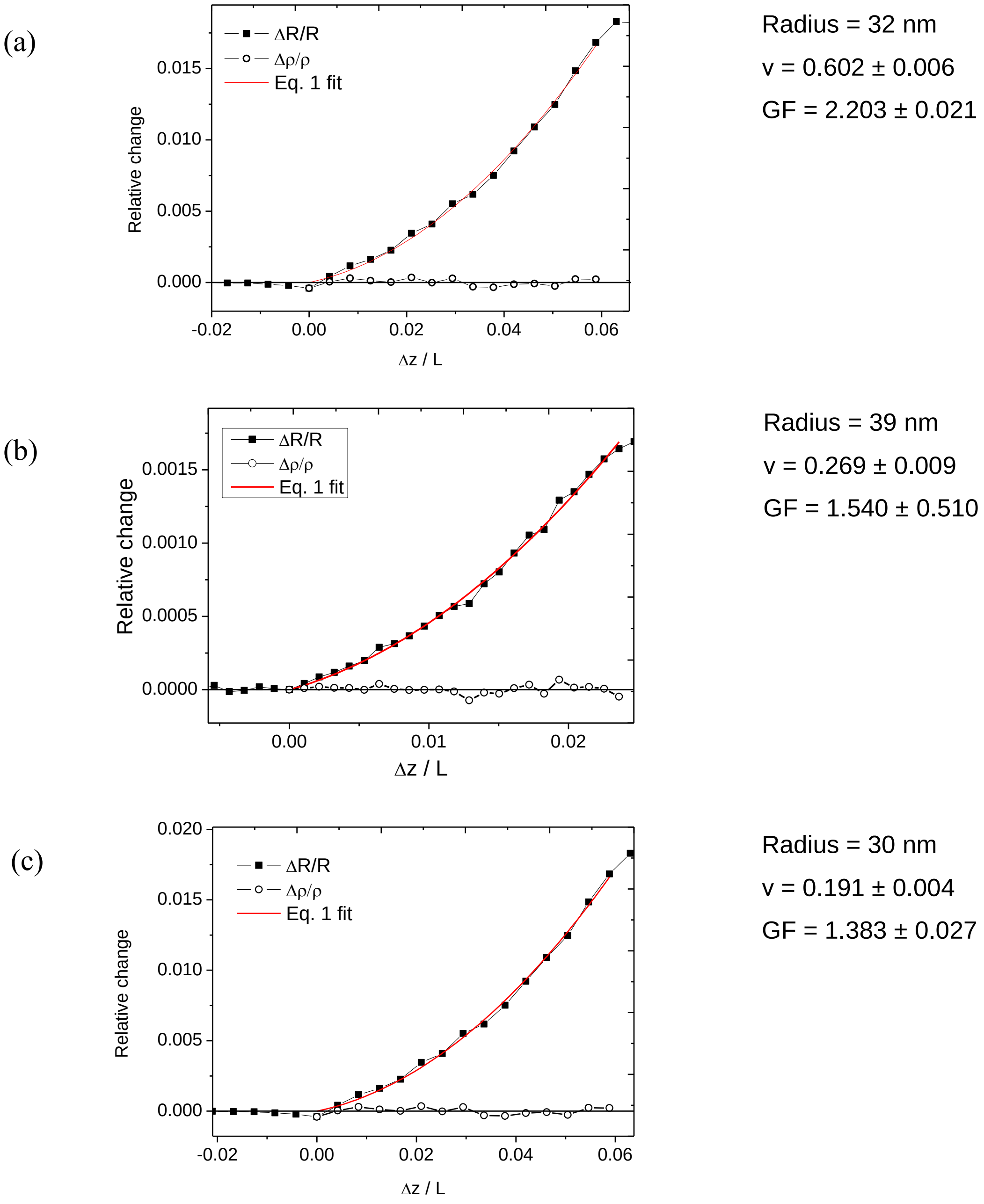

Supplementary Figure 9: Relative change in resistivity and resistance for AgNWs of radii (**a**) 32 nm, (**b**) 39 nm and (**c**) 30 nm. Poisson's ratio and gauge factor given for each AgNW. See supplementary note 5 for further details.

(a)

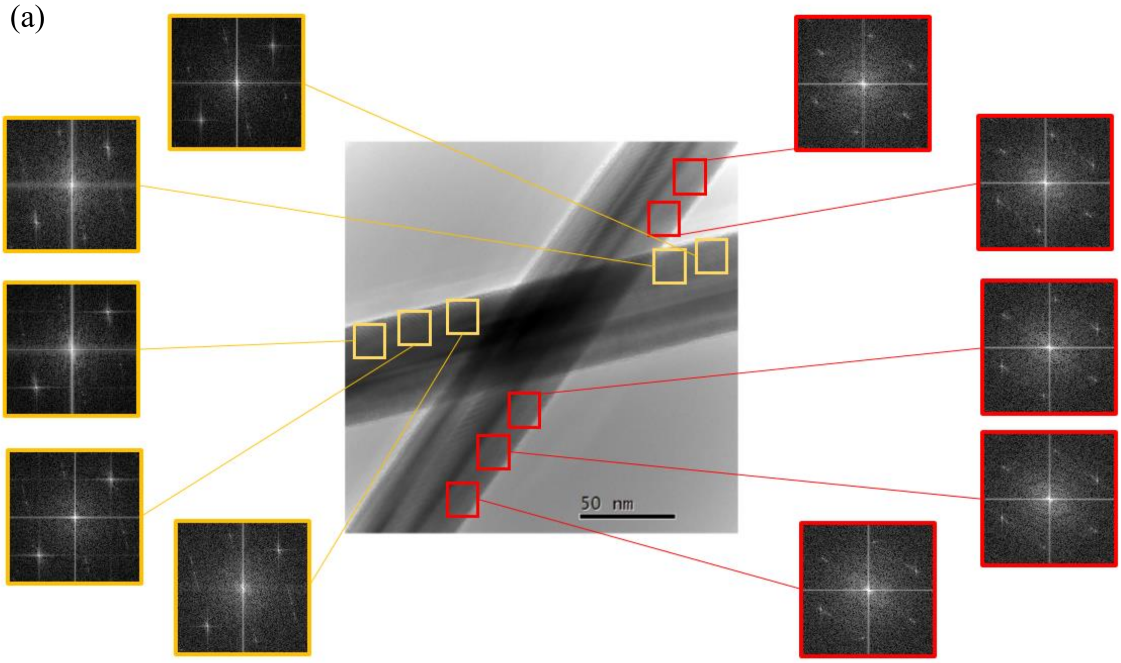

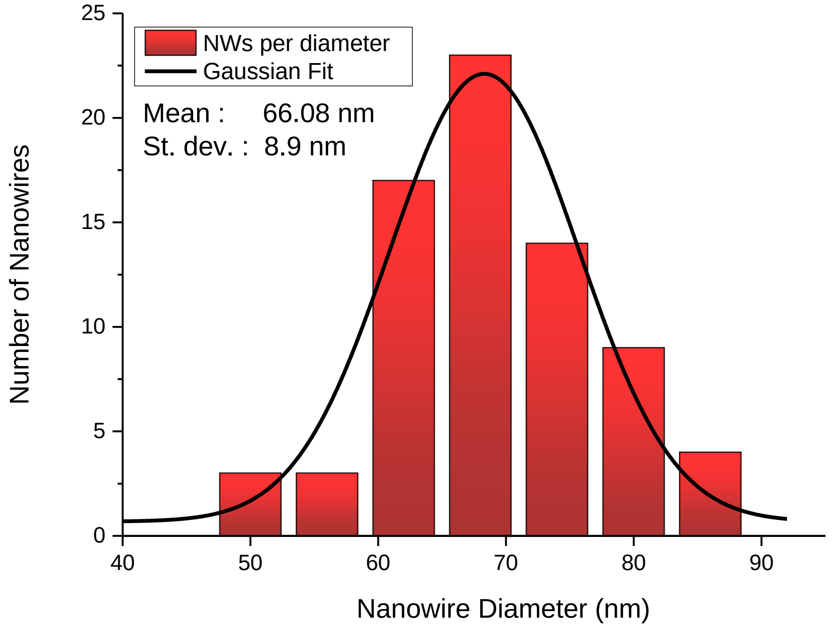

(b)

Supplementary Figure 10: (**a**) TEM image of two AgNWs. The selected area FFTs show there is no change in the NW crystal structure along their length. (**b**) Histogram of NW diameters measured for >70 NWs. The average diameter is 66.08 nm. See supplementary note 6 for further details.

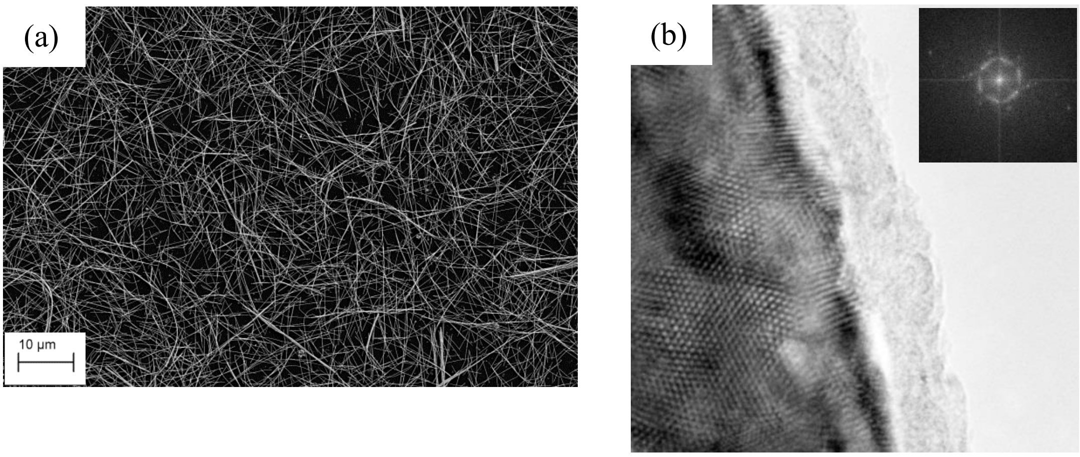

Supplementary Figure 11: (**a**) SEM image of NWs after removal from the AAO template. (**b**) HRTEM image of an individual nickel nanowire. The NW is polycrystalline with a 4 – 8 nm surface oxide. Inset is a FFT showing a typical diffraction pattern for a polycrystalline material. (10 nm scale bar). See supplementary note 6 for further details.

Supplementary Table 1: Detailed outline of all data

		Radius	Loading number	Fit coefficient 2(1+2 ν) Value	Fit coefficient 2(1+2 ν) Standard error	Poisson's ratio ν Value	Poisson's ratio ν Standard error
Macroscopic	Phosphur bronze	62.5 μm	1	3.34882	0.01881	0.337205	0.001894048
	Copper	62.5 μm	1	3.4516	0.06151	0.3629	0.00646714
	Silver	50 μm	1	3.53521	0.07798	0.3838025	0.008465952

	Radius	Loading number	Fit coefficient 2(1+2 ν) Value	Fit coefficient 2(1+2 ν) Standard error	Poisson's ratio ν Value	Poisson's ratio ν Standard error
NiNW	40 nm	1	3.29896	0.05626	0.32474	0.00553807
	44 nm	1	3.218604	0.085614	0.304651	0.008103635
	39 nm	1	3.33845	0.13877	0.3346125	0.013908903
	42 nm	1	3.2651	0.04422	0.316275	0.004283385
	43 nm	1	3.22491	0.25413	0.3062275	0.0241314

	Radius	Current density	Fit coefficient 2(1+2 ν) Value	Fit coefficient 2(1+2 ν) Standard error	Poisson's ratio ν Value	Poisson's ratio ν Standard error
AgNW	32 nm	15.26345	4.4068	0.04198	0.6017	0.005731907
		18.27649	4.46752	0.13463	0.61688	0.018589856
		20.54988	4.59088	0.23897	0.64772	0.033715899
		22.81457	4.15288	0.18857	0.53822	0.024438979
	30 nm	58.08977	2.76532	0.05495	0.19133	0.003801941
		58.08977	2.75821	0.0451	0.1895525	0.003003444
		58.08977	2.78521	0.05333	0.1963025	0.003803194
		58.08977	2.7543	0.09178	0.188575	0.006283779
	39 nm	16.05175	3.0792	0.27519	0.2698	0.089370616
		18.57379	3.2434	0.12399	0.31085	0.038228402
		21.31169	3.25936	0.1368	0.31484	0.04197143
		23.87115	3.28312	0.07769	0.32078	0.023663466
		42.19939	3.08764	0.11315	0.27191	0.036646112
		52.93592	3.04468	0.06351	0.26117	0.020859335
		79.26858	3.06716	0.12283	0.26679	0.040046819
		105.26619	3.35568	0.07393	0.33892	0.022031302
	27 nm	7.1509	2.87863	0.06273	0.2196575	0.004786692
		7.1509	2.81863	0.07876	0.2046575	0.005718674
		7.1509	2.84863	0.08166	0.2121575	0.006081794
		7.1509	2.84754	0.00939	0.211885	0.000698708
		8.9250	2.92271	0.07996	0.2306775	0.006310914
		8.9250	2.90863	0.09449	0.2271575	0.007379458
		8.9250	2.90132	0.09197	0.22533	0.007142818
		8.9250	2.98176	0.07558	0.24544	0.006221277
		10.6991	2.91983	0.12687	0.2299575	0.00999192
		10.6991	2.93937	0.14726	0.2348425	0.011765415

		10.6991	2.91635	0.14687	0.2290875	0.011537052
		10.6991	2.95712	0.15457	0.23928	0.012507274
		12.4707	2.90756	0.15725	0.22689	0.012270926
		12.4707	2.90825	0.21678	0.2270625	0.016925164
		12.4707	2.90654	0.14617	0.226635	0.011397482
		12.4707	2.92426	0.1687	0.231065	0.013330096
		7.1509	2.88069	0.15267	0.2201725	0.01166864
		7.1509	2.89507	0.17155	0.2237675	0.013259546
		7.1509	2.8811	0.13068	0.220275	0.009991162
		7.1509	2.85072	0.19636	0.21268	0.014649578
		8.9250	2.84369	0.1777	0.2109225	0.013180385
		8.9250	2.91658	0.32722	0.229145	0.025708476
		8.9250	2.91674	0.13325	0.229185	0.010470217
		8.9250	2.92951	0.04465	0.2323775	0.003541772
		10.6991	2.89934	0.08245	0.224835	0.006393747
		10.6991	2.91131	0.07798	0.2278275	0.006102404
		10.6991	2.93434	0.10887	0.233585	0.00866648
		10.6991	2.92549	0.05308	0.2313725	0.004198015

Supplementary note 1: AFM length measurement accuracy

The spanning length of the NW between the trench edges i.e. the clamped length, is measured by AFM before and after manipulation. The accuracy of the AFM tip in measuring the NW span length is critical in understanding the error in the measured values of Poisson's ratio. Supplementary figure 3a shows an AFM image of the trench, encapsulating both the bottom of the trench and the trench edges. A line profile recorded along the red line in supplementary figure 3a is shown in supplementary figure 3b. The NW is suspended above the trench in all experiments, hence, the initial drop-off region at the upper edge of the trench is the critical contact area. An enlarged plot of the area within the red square in supplementary figure 3b is shown in supplementary figure 3c. From supplementary figure 3c the AFM accuracy is given by the intersection of the red line plots, point 1, to the data point labelled point 2. The distance is 13 nm and given that there are two edges the overall accuracy is 26 nm. Taking the overall width of the trench as 1884 nm, the accuracy in the measurement is 1.4 %.

Supplementary note 2: Nanowire clamping at the trench edges

In all experiments the NW surface adhesion prior to the trench edge acts to clamp the wire. To prove this a 54 nm diameter AgNW is loaded to fracture and an SEM used to image the area after fracture had occurred. (NOTE: all other measurements are purely in the elastic regime far below the fracture point. This experiment is performed under extreme loading to show slippage does not occur). Supplementary figure 4 shows an SEM image of a 54 nm diameter AgNW. The breaking point of the NW is clearly visible in the centre of the spanning length, the point at which the AFM tip loads the NW. Mild plastic deformation is evident as the NW broken segments remain in the extended state. The red line follows the centre of the NW long axis. The line deviates from the centre of the NW long axis at the trench edge and hence there is no slip beyond the trench edge. To this end we can conclude

that there is no movement of the NW beyond the trench edge during manipulation, even under extreme loading conditions. The bright regions at the edge of the trench are the sloped side walls leading to the bottom of the trench. The side wall slope is ~200 nm from the top to the bottom of the trench, which is clearly evident, and supported by, the AFM line profile in supplementary figure 3b.

Supplementary note 3: AgNW Electromechanical Measurement Fits and Heating Effects

Supplementary figure 3 and 4 show both the mechanical and electromechanical data fit to the relevant models, including the residuals, for both 32 nm and 39 nm AgNWs. Also shown is the change in resistance with time as a function of increasing current loads, demonstrating that no heating effects are observed.

Supplementary note 4: NiNW Electromechanical Data

Supplementary figure 8 shows the relative change in resistivity and resistance as a function of $\Delta z/L$ for several NiNWs referred to in the main text. The same behaviour is observed across all of the wires examined.

Supplementary note 5: AgNW Electromechanical Data

Supplementary figure 9 shows the relative change in resistivity and resistance as a function of $\Delta z/L$ for several AgNWs referred to in the main text. The same behaviour is observed across all of the wires examined.

Supplementary note 6: TEM Analysis of AgNW and NiNW

Supplementary figure 10 displays the rigorous TEM analysis performed on the AgNWs used in this study. Supplementary figure 5 in the main text showed clear evidence of 5-fold

twinning structure in AgNWs. Supplementary figure 10a shows a TEM image of two crossed AgNWs. The FFT images at the selected areas along a considerable length of each NW is analysed and show that the crystal structure does not change along the wire length.

The size dispersion and the change in diameter along the length of the NW contribute to the measured electrical and mechanical response. The diameters of >70 AgNWs are measured , using TEM, and yielded an average diameter of 66.08 nm and a standard deviation of 8.90 nm, shown by the histogram in supplementary figure 10b. To study the variation in the diameter along the length of a given wire the diameter of each NW is measured 15 times along a 2μm length sections. The variation in the diameter along the length of the NW is found to be ± 0.79 nm, following >1500 individual diameter measurements.

Supplementary figure 11a shows a low magnification SEM image of the NiNWs used in this study. From supplementary figure 11a it is clearly seen that the NWs have a high aspect ratio and there are very few agglomerated wires. Supplementary figure 11b shows a HRTEM image of an individual polycrystalline NiNW. The FFT, inset, shows a typical diffraction pattern for a polycrystalline material. A ~4 nm surface oxide, critically important for the observed RS behaviour, is clearly present.

Supplementary Discussion

Relationship between resistance and resistivity changes in a nanowire under normal loading.

Bending

We first consider the contribution due to bending, according to classical Euler-Bernoulli beam theory – this applies to high aspect ratio NW (length/width). All strain components vary linearly with *z* (coordinate normal to the applied load direction). These do not contribute to the relationship between the measured net change in resistance and resistivity because they balance symmetrically across the thickness – their average (total) effect over the beam volume is zero, due to their linear dependence on *z*. In reality, shear deformation will be present for a beam of finite thickness-to-length ratio, and is expected to yield a nonzero contribution. This will induce a net strain of $O\left(\left[h/L\right]^4 \Delta z/h\right)$ where Δz is the beam displacement, *h* its thickness and *L* its length, which for a long and narrow beam will (normally) be smaller than any contribution due to stretching, as we discuss below.

Stretching

As the beam deflects normal to its long axis, strain develops along the beam due to stretching:

$$e_{xx} = \frac{1}{2L}\int_0^L \left(\frac{dw}{dx}\right)^2 dx \qquad (1)$$

where *w* is the deflection normal to the beam axis. This induces strains in two orthogonal directions:

$$e_{yy} = e_{zz} = -\nu e_{xx} \qquad (2)$$

where ν is Poisson's ratio.

It is shown by Ngo et al[1] that the relationship between the induced uniaxial tensile force, T, due to stretching and the centre beam deflection, Δz, is well approximated by:

$$\frac{TL^2}{EI} = \frac{6\varepsilon(140+\varepsilon)}{250+3\varepsilon}, \tag{3}$$

where

$$\varepsilon = \Delta z^2 \left(\frac{A}{I} \right), \tag{4}$$

and I is the areal moment of inertia of the cross section, and A is its area. This formula is accurate to ~2%, in comparison to the exact solution.

From Hooke's law, it follows that the strain along the beam axis is given by:

$$e_{xx} = \left(\frac{I}{AL^2} \right) \frac{6\varepsilon(140+\varepsilon)}{250+3\varepsilon} \tag{5}$$

Equations (2) and (5) completely describe the strain tensor in the beam as all other strain components are zero.

The resistance, R, is related to the resistivity, ρ, by

$$R = \frac{\rho L}{A} \tag{6}$$

It directly follows that the relationship between the change in resistance, ΔR, and the change is resistivity, $\Delta \rho$, is

$$\frac{\Delta R}{R} = \frac{\Delta \rho}{\rho} + \frac{\Delta L}{L} - \frac{\Delta A}{A} \tag{7}$$

This can be written in terms of the strain tensor components:

$$\frac{\Delta\rho}{\rho} = \frac{\Delta R}{R} - e_{xx} + e_{yy} + e_{zz} \tag{8}$$

Substituting equations (2) and (5) into equation (8) results in:

$$\frac{\Delta\rho}{\rho} = \frac{\Delta R}{R} - (1+2\nu)\left(\frac{I}{AL^2}\right)\frac{6\varepsilon(140+\varepsilon)}{240+3\varepsilon} \tag{9}$$

Equation (9) allows the relative change in resistivity, $\Delta\rho/\rho$, to be determined from the measured change in resistance, $\Delta R/R$, the centre displacement and the beam dimensions.

If we consider the case where Δz is much smaller than the beam radius, then equation (9) simplifies:

$$\frac{\Delta\rho}{\rho} = \frac{\Delta R}{R} - \frac{12(1+2\nu)}{5}\left(\frac{\Delta z}{L}\right)^2 \tag{10}$$

Thus, the resistance and resistivity are related by the square of the beam displacement. If Δz is greater than the beam radius, i.e. in the case of large beam deflection, we again obtain a quadratic dependence with strain, albeit with a slightly different coefficient:

$$\frac{\Delta\rho}{\rho} = \frac{\Delta R}{R} - 2(1+2\nu)\left(\frac{\Delta z}{L}\right)^2 \tag{11}$$

Shear deformation induces a correction of higher order in the thickness-to-length ratio correction, however, this should only be observable at very small deflections, i.e.

$$\frac{\Delta z_{centre}}{a} < \left(\frac{a}{L}\right)^2 \tag{12}$$

Measurements are performed well away from this limit, so shear deformation effects does not affect the theory.

Young's Modulus Measurement

Supplementary figure 1 shows a typical force-displacement curve for an AgNW. The elastic modulus is found to be 86 ± 10 GPa for this 90 nm diameter nanowire, calculated form the well-known generalised model[2];

$$F_{center} = \frac{192EI}{L^3} f(\alpha)\Delta z \qquad (13)$$

where F is the applied force, E is Young's modulus, I is the areal moment of inertia, L is the length, Δz_{center} is the lateral displacement and the $f(\alpha)$ term accounts for tensile deformation along the nanowire. A full description of the mechanical model is described elsewhere[2].

Generally, we found E to be between 40 - 160 GPa for individual pentagonal AgNWs agreeing with previously reported values[3-5]. Supplementary figure 2 shows a typical 4-point IV for an individual suspended pentagonal AgNW, the inset shows both 2- and 4-point measurements. As expected the 2-point measurement is dominated by the contact resistance. The NW is ohmic with a resistance, R = 15.5 Ω. In general, R ~15 -70 Ω, depending on the diameter and length of the NW, with ρ ~ 17 – 35 nΩ m. The NW resistivity is found to increase with decreasing radius, consistent with the general trend in the literature [6]. In addition, the resistance and resistivity values agree with previously reported measurements on similar nanowires[7].

NiNW Electrical Preparation: a Resistive Switching (RS) Process

NiNWs display a physical phenomenon known as resistive switching (RS), so that a number of electrical forming steps must be performed before any 4-point measurements are possible.

The forming steps produce a conductive filament (CF), through the NiO layer that coats the wire, resulting in an ohmic connection between the Ni core and the contacting electrode. The RS process has been described extensively elsewhere[8-10].

Supplementary figure 7 outlines the CF forming process. Supplementary figure 7a shows a schematic of the NW contacted with four blue electrodes labelled 1-4. Firstly, the area designated as “FORM 1” is made conductively active across electrodes 1 and 2 by the forming process, given by the IV curve in supplementary figure 7b. Between 0 – 2.8 V the NW is in the high-resistance state as there are no CFs at the NW/contact-1 or NW/contact-2 junctions. At a threshold voltage of 2.8 V the resistance has an abrupt decrease to the low resistance state. Here, the current is limited by a set compliance current of 500 nA. The NW remained in the low resistance state, signifying that a non-volatile CF had formed. The same procedure is applied to the “FORM 2” section between electrodes 3 and 4, given by the IV curve in supplementary figure 7c. We see the same characteristics as supplementary figure 7b but with a higher compliance current of 10 μA. The higher compliance current is necessary for the NW to remain in the high resistance state at 0 V, more than likely due to a slightly thicker oxide at one, or both, electrodes 3 and 4. After the forming process standard 4-point electrical characterisation could be performed, given by the 2- and 4-point IV curve shown in supplementary figure 7d. The voltage is sourced and the current is measured across the outer electrodes 1 and 4, the voltage is measured across the inner electrodes 2 and 3. The IV curve displays typical linear behaviour observed in metals, showing that the forming process produces secure metallic filaments between the Ag electrode and NiNW core. Typically the 4-point resistance is found to be between 60 – 200 Ω, depending on the dimensions of the NW, with resistivities between 70 – 190 nΩ m comparable with the bulk value (69 nΩ m).

Supplementary References

1. Ngo, L.T., et al., *Ultimate-Strength Germanium Nanowires.* Nano Letters, 2006. **6**(12): p. 2964-2968.
2. Heidelberg, A., et al., *A Generalized Description of the Elastic Properties of Nanowires.* Nano Letters, 2006. **6**(6): p. 1101-1106.
3. Bin Wu, A.H., John J. Boland, John E. Sader, XiaoMing Sun, YaDong Li, *Microstructure-Hardened Silver nanowires.* Nanoletters, 2006. **6**(3): p. 468-472.
4. Jing, G.Y., et al., *Surface effects on elastic properties of silver nanowires: Contact atomic-force microscopy.* Physical Review B, 2006. **73**(23): p. 235409.
5. Li, X., et al., *Nanoindentation of Silver Nanowires.* Nano Letters, 2003. **3**(11): p. 1495-1498.
6. Kuhn, K.J., *Considerations for Ultimate CMOS Scaling.* Electron Devices, IEEE Transactions on, 2012. **59**(7): p. 1813-1828.
7. Kolesnik, M.M., et al., *Resolving In Situ Specific-Contact, Current-Crowding, and Channel Resistivity in Nanowire Devices: A Case Study with Silver Nanowires.* Small, 2011. **7**(20): p. 2873-2877.
8. Kwon, D.-H., et al., *Atomic structure of conducting nanofilaments in TiO2 resistive switching memory.* Nat Nano, 2010. **5**(2): p. 148-153.
9. Park, G.-S., et al., *Observation of electric-field induced Ni filament channels in polycrystalline NiO[sub x] film.* Applied Physics Letters, 2007. **91**(22): p. 222103-3.
10. Kim, S.I., et al., *Reversible resistive switching behaviors in NiO nanowires.* Applied Physics Letters, 2008. **93**(3): p. 033503-3.
